# De novo aggregation of Alzheimer’s A*β*25-35 peptides in a lipid bilayer

**DOI:** 10.1038/s41598-019-43685-7

**Published:** 2019-05-09

**Authors:** Amy K. Smith, Dmitri K. Klimov

**Affiliations:** School of Systems Biology, George Mason University, Manassas, VA 20110 USA

**Keywords:** Membrane biophysics, Computational biology and bioinformatics

## Abstract

A potential mechanism of cytotoxicity attributed to Alzheimer’s A*β* peptides postulates that their aggregation disrupts membrane structure causing uncontrollable permeation of Ca^2+^ ions. To gain molecular insights into these processes, we have performed all-atom explicit solvent replica exchange with solute tempering molecular dynamics simulations probing aggregation of the naturally occurring A*β* fragment A*β*25-35 within the DMPC lipid bilayer. To compare the impact produced on the bilayer by A*β*25-35 oligomers and monomers, we used as a control our previous simulations, which explored binding of A*β*25-35 monomers to the same bilayer. We found that compared to monomeric species aggregation results in much deeper insertion of A*β*25-35 peptides into the bilayer hydrophobic core causing more pronounced disruption in its structure. A*β*25-35 peptides aggregate by incorporating monomer-like structures with stable C-terminal helix. As a result the A*β*25-35 dimer features unusual helix head-to-tail topology supported by a parallel off-registry interface. Such topology affords further growth of an aggregate by recruiting additional peptides. Free energy landscape reveals that inserted dimers represent the dominant equilibrium state augmented by two metastable states associated with surface bound dimers and inserted monomers. Using the free energy landscape we propose the pathway of A*β*25-35 binding, aggregation, and insertion into the lipid bilayer.

## Introduction

Amyloid-*β* (A$\beta$) peptides are the natural products of cellular proteolysis resulting from cleavage of transmembrane amyloid precursor proteins (APP) by *β* and *γ* secretases. Decades of research have established that these peptides play a central role in the onset and development of Alzheimer’s disease (AD), a neurodegenerative condition leading to memory loss and cognitive disfunction. An important physical feature of AD is an appearance of extracellular neuritic plaques or amyloid fibrils composed of aggregated A*β* peptides. These remarkably ordered aggregates rich in *β*-structure^1,2^ predominantly involve two, 40- and 42-residue, peptide species, A*β*1-40 and A*β*1-42. Being derived from transmembrane and extracellular regions of APP, these amyloidogenic peptides contain a highly polar N-terminus, a mixed polar/apolar middle section, and an exclusively hydrophobic C-terminus. Importantly, they demonstrate high *in vitro* and *in vivo* cytotoxicity against various cells including neurons and are capable of degrading intercellular synapses^3–6^. Traditionally, A*β* fibrils were considered the primary AD neurotoxic species, but recent studies probing correlations with dementia symptoms have pointed to soluble oligomers as the most potent cytotoxic A*β* forms^7,8^. Aggregation of A*β* peptides represents the core of the amyloid cascade hypothesis^9^, which postulates that A*β* aggregated species trigger a variety of biochemical pathways eventually leading to neuronal death. Among those are direct interactions of A*β* peptides with neuronal membranes or their binding to microglial and neuronal cellular receptors causing oxidative stress, inflammatory response, and altered calcium homeostasis.

**Figure 1:**
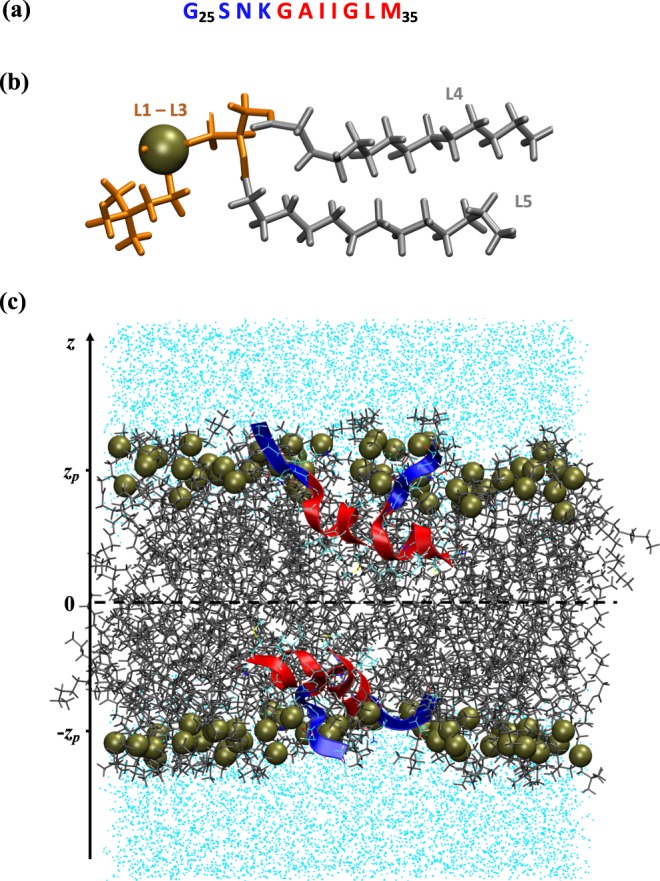
Simulation model for investigating A*β*25-35 aggregation in the DMPC lipid bilayer. (**a**) The sequence of A*β*25-35 peptide. N-terminal R3 and C-terminal R4 regions are shown in blue and red, respectively. (**b**) DMPC lipid consists of five structural groups (see Methods). The polar lipid headgroups L1–L3 are shown in orange, and the fatty acid tails L4 and L5 constituting the hydrophobic core are shown in grey. Phosphorus atom is represented as a tan sphere. (**c**) A snapshot of the DMPC bilayer with two inserted A*β*25-35 dimers in an **ID** state (see Results). Lipids and water are in grey and cyan, respectively, and phosphorus atoms are displayed as tan spheres. R3 (blue) and R4 (red) regions in A*β*25-35 peptides are distinguished. The centers of mass of phosphorus atoms in each leaflet occur, on average, at ±*Z*_*P*_ (*z*_*P*_ = 17.37 Å).

A spectrum of *in vivo* A*β* species is remarkably diverse and includes various N- and/or C-termini truncated peptides^10^. One of them is an 11-mer peptide fragment A*β*25-35 shown in Fig. 1a, which is composed of APP regions embedded in the membrane (29–35) and exposed to the membrane-water interface (25–28)^11^. *In vivo* A*β*25-35 is localized in neurons of the subiculum and entorhinal cortex^12^. This peptide represents an apparent functional domain of the full-length A*β* responsible for its amyloidogenic and cytotoxic properties^3,13^. Consequently, A*β*25-35 is the shortest A*β* fragment retaining some of the amyloidogenic and cytotoxic properties of the full-length peptide^11,14–16^. In particular, A*β*25-35 demonstrates remarkable speed of aggregation and almost immediate, without aging, cytotoxicity *in vitro*^3,13^. For example, fresh A*β*25-35 peptides form sediments within an hour, which is even faster than A*β*1-42^3^, whereas mature A*β*25-35 fibrils have been reported to appear within 12 hours after incubation^17^. Interestingly, rapid development of cytotoxicity may implicate the A*β*25-35 monomers or small oligomers. Indeed, monomeric A*β*25-35 is known to produce apoptotic signals leading to cellular death^15^. Behavioral studies have shown that A*β*25-35 peptides cause amnesia and memory deficits in mice models^18,19^. Because of its small size and properties reminiscent of the full-length peptides, A*β*25-35 has been a target of numerous experimental and *in silico* investigations^15,20–23^.

Interactions of A*β* peptides with cellular membranes may represent one of the primary mechanisms of their cytotoxicity^24–29^. Consequently, binding of A*β*25-35 peptides to lipid bilayers has been extensively examined experimentally. In particular, calorimetric studies revealed strong affinity of A*β*25-35 peptides to anionic POPC/POPG lipid bilayers^30^, whereas neutron diffraction data indicated that A*β*25-35 penetrates and structurally perturbs POPC/POPS bilayers^31^. Notably, the extent of bilayer structural distortion has reportedly exceeded even that observed for the full-length A*β*1-40 or A*β*1-42 peptides. A*β*25-35 binds not only to anionic, but also to zwitterionic lipid bilayers. Electron paramagnetic resonance studies have found that this peptide becomes inserted into the DLPC bilayer, positioning at the boundary between the hydrophobic core and hydrophilic headgroup region^32^. Similar conclusions have been reached in an earlier X-ray diffraction investigation^33^.

Although experimental techniques are indispensable for studying the interactions of A*β*25-35 with cellular membranes, they cannot resolve underlying atomistic details. Nonetheless, this information is critical for understanding the mechanisms of A*β* aggregation within the lipid environment and disruption of bilayer structure, which together are likely to lead to A*β* cytotoxicity. To gain insights into associated molecular mechanisms, we have used replica exchange with solute tempering (REST) simulations to investigate interactions of A*β*25-35 monomer with a DMPC lipid bilayer^34^. We discovered that the monomer binds to the membrane adopting two coexisting states: a stable state bound to the surface polar headgroups and a less stable state embedded in the hydrophobic core. A moderate free energy barrier separates both states, and it is therefore likely that the A*β*25-35 monomer frequently transitions between surface-bound and inserted conformations. Although in the inserted state the peptide induces considerable bilayer disruption, the overall effect on the DMPC bilayer integrity is minimal due to the dominance of surface bound state. In this paper, we extend our previous all-atom REST investigations to probe the aggregation of A*β*25-35 peptides within the DMPC bilayer (Fig. 1). We show that A*β*25-35 peptides readily aggregate into dimers and in contrast to monomeric species penetrate deep into the DMPC bilayer causing extensive damage to its structure. By computing the free energy landscape we reconstruct the pathway of A*β*25-35 binding and aggregation.

## Results

### Aggregation does not substantially reorganize A*β*25-35 structure

**Figure 2:**
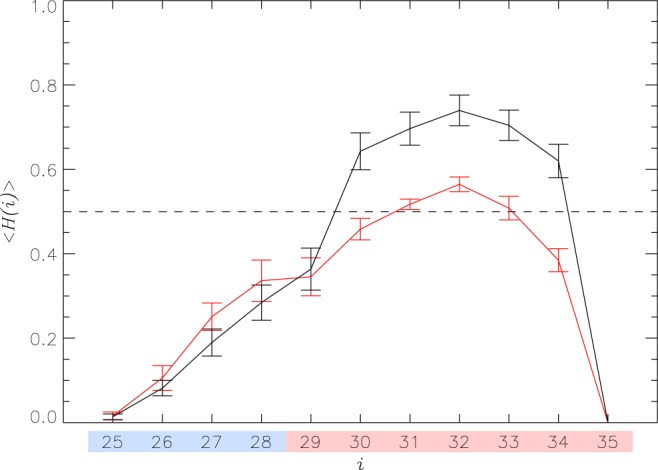
Helical propensities 〈*H*(*i*)〉 for A*β*25-35 amino acids *i*. Data for A*β*25-35 dimers and monomers^34^ are shown in black and red, respectively. Vertical bars show sampling errors. Regions R3 and R4 are colored according to Fig. 1a.

We first investigated the changes in the secondary structure of A*β*25-35 peptides caused by aggregation. (It is important to make a note about terminology. Because dimeric states occur with the probability of 0.64 ± 0.07 (see Models and Methods), for brevity we collectively refer to the peptides sampled in our simulations as dimers unless we specifically distinguish dimeric and monomeric subpopulations). Figure 2 presents the helical propensities 〈*H*(*i*)〉 for amino acids *i* in A*β*25-35 dimers binding to the DMPC bilayer. As a control, we use 〈*H*(*i*)〉 computed in our previous REST simulations probing binding of A*β*25-35 monomers to the same bilayer^34^. Aggregation promotes helical structure increasing the number of amino acids adopting stable helix (〈*H*(*i*)〉 > 0.5) from three in A*β*25-35 monomers to five in the dimers. Overall, A*β*25-35 helix content 〈*H*〉 increases from 0.31 ± 0.03 to 0.39 ± 0.03, although a more pronounced rise is seen in the C-terminal R4 region (from 0.39 ± 0.02 to 0.54 ± 0.02). The analysis of *β*-turn and random coil structure is given in Supplementary Information. Thus, aggregation moderately increases helical propensity, particularly in the C-terminus, with concurrent reduction in *β*-turn conformations, whereas random coil propensity remains largely unaffected. Aggregation causes A*β*25-35 extension manifested in the increase of the end-to-end distance *r*_1*N*_ from 14.4 ± 0.4 to 15.4 ± 0.4 Å. Computation of intrapeptide contacts in Supplementary Information shows that aggregation stabilizes few local interactions, particularly Gly29-Ile32 and Gly29-Gly33, reflecting the enhancement of helical structure. Nonetheless, taken together, the analysis of secondary and tertiary structure does not reveal significant structural reorganization in A*β*25-35 peptides bound to the DMPC bilayer caused by aggregation.

### Aggregation promotes deeper penetration of A*β*25-35 into the DMPC bilayer

**Figure 3:**
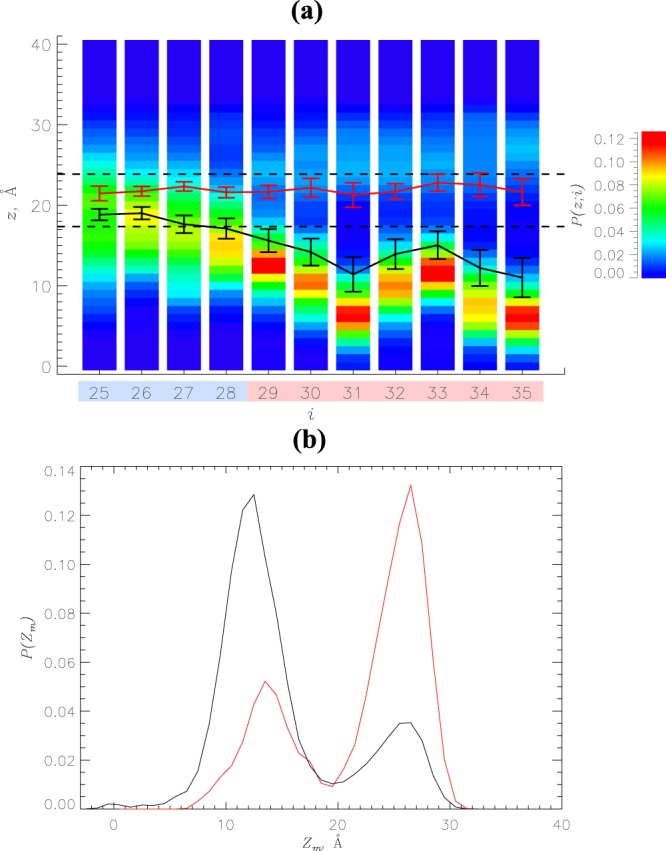
Binding of A*β*25-35 dimers to the DMPC bilayer. (**a**) Probabilities *P*(*z*; *i*) for amino acids *i* in A*β*25-35 dimers to occur at the distance *z* from the bilayer midplane. The scale on the right color codes *P*(*z*; *i*). Black and red lines represent the average positions of amino acids in A*β*25-35 dimers and monomers^34^, 〈*z*(*i*)〉, with sampling errors. The dashed line at *z*_*P*_ separates the bilayer hydrophobic core from its surface region, whereas the dashed line at *z*_*p*_ + 6.5 Å distinguishes the bilayer surface region and solvent. Regions R3 and R4 are colored according to Fig. 1a. (**b**) Probability distributions *P*(*Z*_*m*_) of the positions of the center of mass of A*β*25-35 peptides *Z*_*m*_ along the bilayer normal. Peptide inserted and surface bound states are separated by the minimum midpoint in bimodal *P*(*Z*_*m*_) distributions. Data in black and red correspond to A*β*25-35 dimers and monomers.

Enhanced cytotoxicity of A*β* oligomers can be related to their deeper insertion into lipid bilayers compared to A*β* monomers. To investigate this possibility, we have computed the probabilities *P*(*z*; *i*) for amino acids *i* to occur at a distance *z* from the bilayer midplane. Their distribution shown in Fig. 3a suggests that most amino acids in A*β*25-35 dimer are inserted in the DMPC bilayer. To substantiate this observation, we computed the average locations of amino acids *i* along the bilayer normal, 〈*z*(*i*)〉, and compared them between A*β*25-35 dimers and monomers^34^. With the exception of first three N-terminal amino acids all others in the dimer are inserted into the bilayer, because they occur, on average, below the position of the center of mass of phosphorous atoms, i.e., 〈*z*(*i*)〉 < *z*_*P*_. In contrast, all amino acids in A*β*25-35 monomers reside within the bilayer headgroup region (*z*_*p*_ < *z* < *z*_*P*_ + 6.5 Å), i.e., they are classified as surface bound.

Amino acid positions in the bilayer analyzed above suggest different binding propensities of A*β*25-35 dimers and monomers. To check this assertion, we computed the probability distributions *P*(*Z*_*m*_) of the position of the center of mass of A*β*25-35 peptide *Z*_*m*_ along the bilayer normal. The distributions *P*(*Z*_*m*_) computed for the dimers and monomers presented in Fig. 3b are bimodal. The peptides from A*β*25-35 dimer are inserted in the bilayer with the probability of 0.79 ± 0.08, while the probability of binding to the bilayer surface is only 0.21 ± 0.08. For A*β*25-35 monomers the respective probabilities are almost opposite being 0.31 ± 0.07 and 0.69 ± 0.07^34^. Thus, aggregation dramatically shifts the distribution of A*β*25-35 states from predominantly surface bound for the monomers to overwhelmingly inserted for the dimers. The dependence of A*β*25-35 helical propensity on insertion depth is analyzed in Supplementary Information.

To map residue interactions with lipids, we computed the contact map 〈*C*_*l*_(*i*, *k*)〉, which reports the formation of contacts between amino acids *i* and lipid groups *k*. Figure S4a shows that in the A*β*25-35 dimers the N-terminal R3 amino acids bind exclusively to the DMPC headgroups L1–L3. In contrast, the C-terminal R4 amino acids Ile31, Ile32, Leu34, and Met35 mostly interact with the fatty acid tails L4 and L5, whereas Gly29 and Gly33 exclusively bind to the headgroups. This pattern of amino acid-lipid interactions reflects the formation of C-terminus helix, in which the Gly-rich face of the helix is positioned toward the DMPC headgroups and water as seen in Fig. 3a. For comparison, the contact map 〈*C*_*l*_(*i*, *k*)〉_*m*_ for A*β*25-35 monomers^34^ shown in Fig. S4b demonstrates that binding interactions are largely restricted to the peptide N-terminus and DMPC headgroups. Analysis of the difference in the number of contacts with lipids per amino acid 〈Δ*C*_*l*_(*i*)〉 shown in Fig. S4c leads to two observations. First, aggregation strengthens all amino acid - lipids interactions resulting in the net increase of the number of peptide-bilayer contacts from 12.3 ± 1.1 to 18.9 ± 1.6. Second, the amino acids displaying the largest gains in interactions with the DMPC bilayer are Lys28 ($\langle {\rm{\Delta }}{C}_{l}(28)\rangle =0.9\pm 0.2$), Ala30 (0.9 ± 0.3), Val34 (0.9 ± 0.2), Asn27 (0.7 ± 0.2), and Met35 (0.6 ± 0.2). As a result among all amino acids cationic Lys28 binds most tightly to the DMPC bilayer, i.e., it forms the largest number of contacts with lipids ($\langle {C}_{l}(28)\rangle =2.5\pm 0.2$). In summary, aggregation promotes (i) hydrophobic contacts of A*β*25-35 C-terminus with fatty acid tails and (ii) electrostatic interactions between cationic Lys28 and the anionic lipid phosphate group.

### Aggregation facilitates disruption of DMPC bilayer

**Figure 4:**
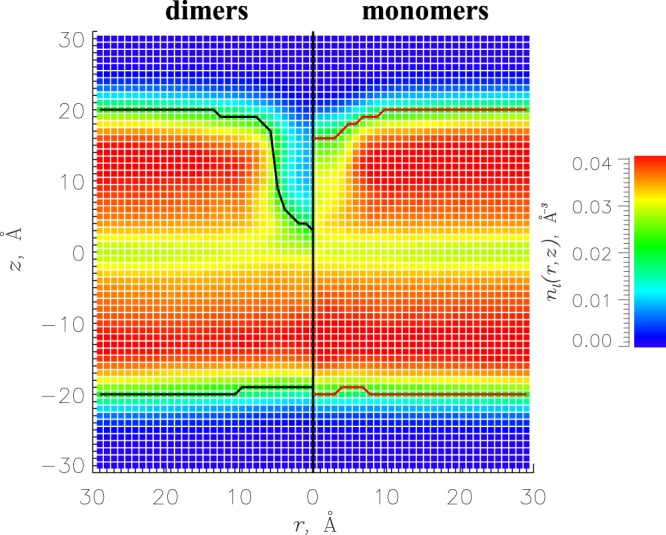
The number density of DMPC heavy atoms *n*_*l*_(*r*, *z*) as a function of the distance *r* to the peptide center of mass and the distance *z* to the bilayer midplane. The density cross-sections for A*β*25-35 dimers and monomers^34^ are given on the left and right sections of the panel. Continuous black and red lines mark the bilayer boundaries *z*_*b*_(*r*). The figure shows that A*β*25-35 dimer induces a deep lipid density void as opposed to A*β*25-35 monomers.

If aggregation promotes deeper penetration of A*β*25-35 peptides into the DMPC bilayer, it is then expected to enhance bilayer disruption. To examine changes in the bilayer structure occurring in response to A*β*25-35 dimer binding, we plot in Fig. 4 the number density *n*_*l*_(*r*, *z*) of DMPC heavy atoms as a function of the distance *r* to the peptide center of mass and the distance *z* to the bilayer midplane. The cross-sectional profile *n*_*l*_(*r*, *z*) shows a deep lipid density void created by aggregated A*β*25-35 species, which is muted for A*β*25-35 monomers^34^. To quantify aggregation impact, we compared the bilayer thickness *D* in the distant and proximal regions (see Models and Methods for definitions). Due to binding of A*β*25-35 monomer the DMPC bilayer thins by Δ*D* = 4.0 ± 1.5 Å^34^. In contrast, binding of A*β*25-35 dimers increases Δ*D* more than four-fold to 17.0 ± 0.9 Å. To illustrate the drop in lipid density, we compared the DMPC surface number densities *n*_*s*_ in the distant and proximal regions. Monomer binding decreases *n*_*s*_ by one-third, from 0.015 ± 0.000 Å^−2^ to 0.010 ± 0.001 Å^−2^ ^34^. In contrast, the corresponding decrease caused by A*β*25-35 dimer is three-fold, from 0.015 ± 0.000 Å^−2^ to 0.005 ± 0.001 Å^−2^. Similar conclusion follows from the analysis of volume number density of lipid heavy atoms *n*_*l*_, which, in response to A*β*25-35 monomer binding, decreases about 25% from 0.034 ± 0.000 in the distant region to 0.025 ± 0.001 Å^−3^ in the center of binding footprint. For comparison, corresponding changes caused by A*β*25-35 dimer are almost three-fold, from 0.034 ± 0.000 to 0.013 ± 0.002 Å^−3^. We show in Supplementary Information that A*β*25-35 dimer has a stronger disordering impact on DMPC fatty acid tails than monomeric peptides. Thus, taken together A*β*25-35 dimers cause a strikingly stronger disruption in the bilayer structure than the monomers.

### Free energy landscape of A*β*25-35 aggregation

**Figure 5:**
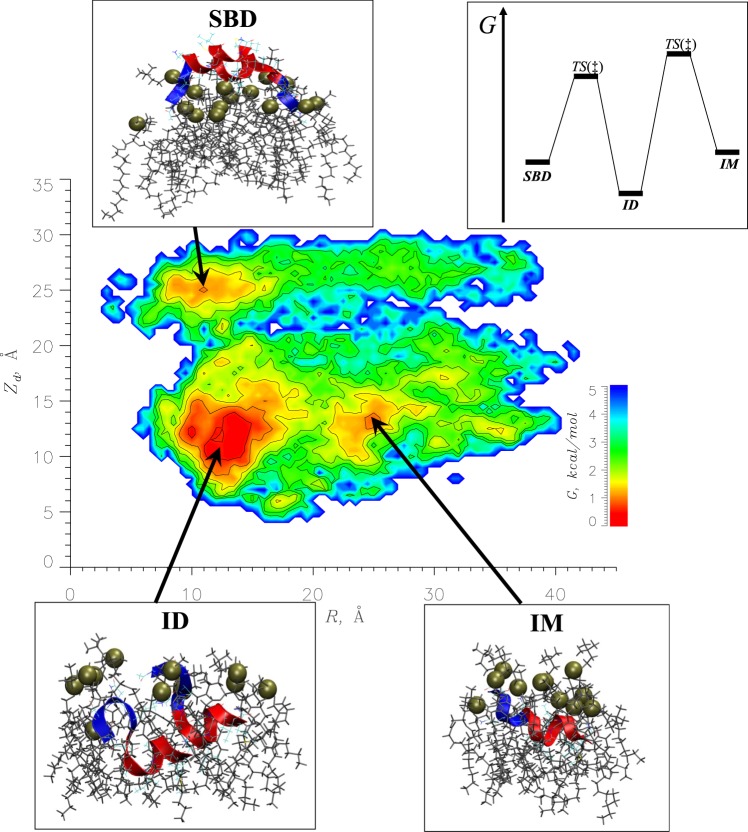
The free energy of A*β*25-35 dimers *G*(*Z*_*d*_, *R*) as a function of the distance *R* between the peptides’ centers of mass and the distance *Z*_*d*_ between the dimer center of mass and the bilayer midplane. The contours are drawn with the 0.5 kcal/mol increment for the free energies *G* ≤ 5 kcal/mol. Three low free energy states, **ID**, **SBD**, and **IM**, are identified and the representative structures from their most populated conformational clusters are shown. The colors of R3 and R4 regions follow Fig. 1a. Lipids with phosphorous atoms represented by tan spheres, which surround A*β*25-35 peptides, are shown. The diagram summarizing the low free energy and transition states included in Table 1 is shown in the upper right corner.

A mechanism of A*β*25-35 aggregation and binding to the DMPC bilayer can be gleaned from the free energy landscape. To this end, we computed the free energy of A*β*25-35 peptides $G({Z}_{d},R)=-\,RT\,{ln}\,P({Z}_{d},R)$ using the probability $P({Z}_{d},R)$ to observe A*β*25-35 peptides with their centers of mass separated by the distance *R* and the center of mass of both peptides located at the distance *Z*_*d*_ from the bilayer midplane. The resulting free energy landscape in Fig. 5 reveals three distinct basins or states listed in Table 1. The lowest free energy state **ID** is associated with A*β*25-35 dimers inserted into the bilayer (*Z*_*d*_ ~ 12 Å and *R* ~ 12 Å). The probability for A*β*25-35 peptides to participate in **ID** is 0.55 ± 0.7. The second state **SBD** separated from **ID** by the free energy gap Δ*G* = 0.8 kcal/mol represents A*β*25-35 dimers bound to the bilayer surface (*Z*_*d*_ ~ 25 Å and *R* ~ 11 Å). This state has the probability of 0.12 ± 0.05. Finally, the third state **IM** separated from **ID** by the free energy gap Δ*G* = 0.9 kcal/mol is populated by the dissociated A*β*25-35 peptides inserted in the bilayer (*Z*_*d*_ ~ 13 Å and *R* ~ 25 Å). The probability of **IM** is 0.11 ± 0.05. The free energy barriers separating the three states are given in Table 1. Their values indicate that the inserted dimers **ID** are surrounded by the highest free energy barriers (Δ${G}^{\dagger }\simeq 4\,{\rm{kcal}}$/mol), whereas the inserted monomers **IM** and surface bound dimers **SBD** reside in more shallow free energy basins (Δ${G}^{\dagger }\simeq 3\,{\rm{kcal}}$/mol). Notably, there is no direct low free energy path in Fig. 5 between **IM** and **SBD** states, which may interconvert only by passing through **ID** serving as intermediate. To provide structural description of states we analyzed A*β*25-35 structures used in computing the free energies of states (Table 1).

**Table 1:** A*β*25-35 low free energy states.

State k	*P*(*k*)^*a*^	*G*(*k*)^*b*^, kcal/mol	k → l^*c*^	*G*^†^, kcal/mol
**ID**	0.55 ± 0.07	0.0 ± 0.2	**ID** → **IM**	4.2
			**ID** → **SBD**	3.7
**SBD**	0.12 ± 0.05	0.8 ± 0.5	**SBD** → **ID**	2.9
**IM**	0.11 ± 0.05	0.9 ± 0.3	**IM** → **ID**	3.3

^a^Fraction of A*β*25-35 peptides included in a state **k**. To compute *P*(*k*) we considered all structures in a basin with the free energies $[{G}_{k,{\min }},{G}_{k,\,{\min }}^{\dagger }]$, where *G*_*k*,*min*_ is the minimum free energy in **k** and ${G}_{k,\,{\min }}^{\dagger }$ is the free energy of transition state along the minimum free energy path from **k**.

^b^To compute the free energy of **k**, *G*(*k*), we integrated *G*(*Z*_*d*_, *R*) within the interval [*G*_*k*,*min*_, *G*_*k*,*min*_ + 0.5 *kcal*/*mol*].

^c^Transition from state **k** to state **l**.

#### Inserted dimers

**Figure 6:**
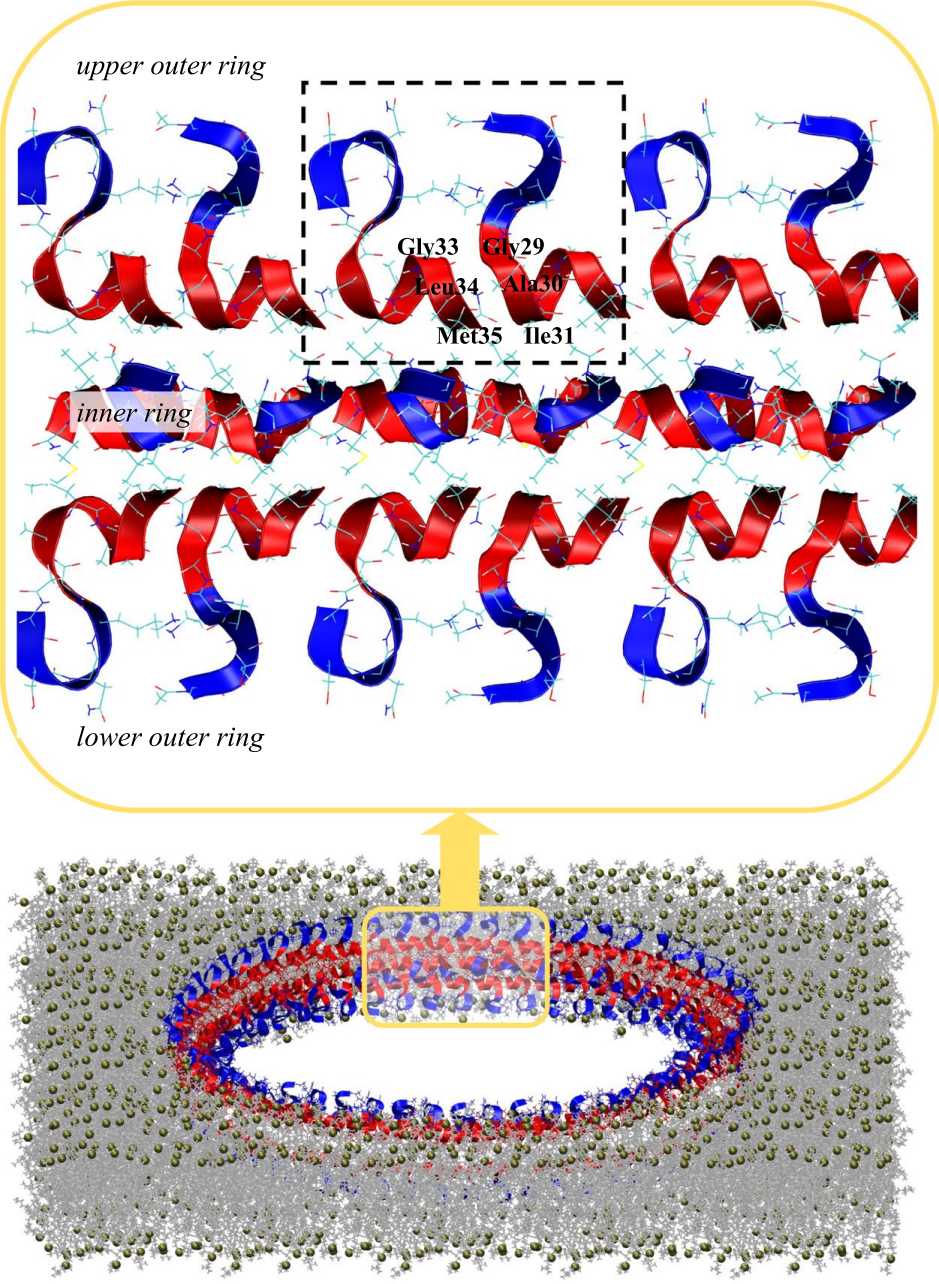
A hypothetical structure of A*β*25-35 annular oligomer or pore in the lipid bilayer formed by three concentric rings of peptides. There are two outer rings placed in the upper and lower bilayer leaflets interacting via their hydrophobic C-termini (in red) with the bilayer core. The peptides N-termini (in blue) occur next to the bilayer surface interacting with the lipid headgroups. The third inner ring is shifted closer to the pore center and its polar N-termini are directed toward the pore center creating its polar lining. The inset magnifies the six peptides from each of the three rings. As building blocks the rings utilize **ID** A*β*25-35 dimers enclosed by dashed lines. The amino acids participating in the dimer parallel aggregation interface are identified. The bilayer representation follows that used in Fig. 1c. The oligomer has a diameter of 20 nm as measured by AFM^36^.

Clustering of **ID** dimers with the cut-off *R*_0_ = 4.4 Å (see Models and Methods) revealed one dominant cluster *ID1* shown in Fig. 5, which includes 86% of **ID** dimers. The distinctive feature of *ID*1 is the formation of parallel out-of-registry aggregation interface composed of the sequence regions Gly33-Met35 and Gly29-Ile31, which are linked by three stable (>0.40) hydrophobic interpeptide amino acid contacts - Gly29-Gly33 ($\langle C(29,33)\rangle =0.87$), Ala30-Leu34 (0.69), Ile31-Met35 (0.50). In addition, *ID1* aggregation interface involves, on an average, 1.7 backbone hydrogen bonds. Importantly, the helical conformations in *ID*1 peptides predominantly occur in the C-terminal R4 region ($\langle H(R4)\rangle =0.60$), while being sparse in the N-terminus ($\langle H(R3)\rangle =0.13$). Due to such distribution of helical structure, A*β*25-35 peptides in a dimer are arranged in a helix head-to-tail tandem as shown in the inset to Fig. 6. Further analysis of *ID*1 shows that the “idle” aggregation interfaces in the left or right peptides are available to add new peptides. Indeed, the probability that all the three interface amino acids have dangling hydrogen bond donors (or acceptors) is 0.64. Rarely, **ID** dimers populate the second cluster *ID2*, which includes 9% of structures. The aggregation interface of this cluster implicates a T-like arrangement of peptides, in which interactions are formed between R3 region in one peptide and both, R3 and R4, regions in the other. Interestingly, the structures of **ID** A*β*25-35 peptides are similar to the inserted monomers **I** sampled in our previous REST simulations, which studied binding of isolated A*β*25-35 monomers to the DMPC bilayer^34^ (see Supplementary Information).

#### Surface bound dimers

A*β*25-35 dimers **SBD** bound to the bilayer surface and clustered with the cut-off *R*_0_ = 5.1 Å populate two distinct clusters, *SBD*1 and *SBD*2, representing 57 and 31% of **SBD** structures. The distinct feature of the most populated cluster *SBD*1 shown in Fig. 5, which sets it apart from **ID** dimers, is the aggregation interface, in which two helical peptides are docked side-by-side in antiparallel arrangement. This interface is supported by four stable ($\langle C(i,j)\rangle  > 0.4$) hydrophobic side chain contacts - Ile31-Leu34 ($\langle C(31,34)\rangle =0.70$), Ile31-Met35 (0.65), Ile31-Ile31 (0.45), and Ile32-Leu34 (0.43).

#### Inserted monomers

Clustering inserted monomers **IM** with the cut-off *R*_0_ = 2.5 Å produced two populated clusters, *IM1* and *IM2* (Fig. 5), which encompass 67 and 21% of structures. The common feature of **IM** clusters is the formation of stable helical structure in R4, but they differ with respect to the occurrence of stable interactions between R3 and R4 regions (specifically, the contact Asn27-Ala30 is present in IM1 and absent in IM2). Importantly, the characteristics of **IM** are similar to those of inserted monomers **I** sampled in our previous REST simulations^34^ (See Supplementary Information).

## Discussion

Although specific causes of cytotoxicity of A*β* aggregated species remain unknown, several potential mechanisms have been proposed, including enhanced production of oxygen reactive species, activation of toll-like receptors leading to local inflammation, and increased permeation of Ca^2+^ ions through neuronal membranes^35^. The latter mechanism assumes that aggregation of A*β* peptides results in formation of structured pores in the bilayer, which were characterized experimentally^27,36–38^, and/or induces sufficient destabilization in interlipid interactions causing increased ion permeability through transient pores^39^. Conversely, this mechanism implies that monomeric A*β* species are less cytotoxic due to their limited impact on the bilayer structure. Because our all-atom explicit solvent simulations compared the binding of A*β*25-35 monomers^34^ and dimers to the DMPC bilayer, they are well positioned to glean molecular insights into putative mechanisms of cytotoxicity.

First, we have demonstrated that aggregation promotes deeper insertion of A*β*25-35 peptides into the zwitterionic DMPC bilayer. Indeed, all except three N-terminal amino acids in the A*β*25-35 dimer are localized in the bilayer hydrophobic core, whereas all amino acids in the A*β*25-35 monomer are bound to the DMPC bilayer surface. The probability distribution *P*(*Z*_*m*_) of the positions of A*β*25-35 peptide center of mass along the bilayer normal is bimodal due to the coexistence of surface-bound and inserted states. Strikingly, aggregation shifts their distribution away from being predominantly surface bound to overwhelmingly inserted (almost 80%). These results are in good agreement with the available experimental data, which implicate A*β*25-35 binding to uncharged liposomes^33^, model zwitterionic POPC membranes^40^, and zwitterionic DLPC multilamellar vesicles^32^. Furthermore, Dante *et al*.^40^ have found that upon binding to the POPC bilayer A*β*25-35 at 3 mol% concentration resides in two states as detected by the location of deuterated D-Leu34. It is notable that in 86% of A*β*25-35 states this residue is positioned 14 Å away from the bilayer midplane, which is consistent with the distance of 13 Å predicted by our simulations (Fig. 3a). In remaining 14% of states D-Leu34 is located at the distance of 27 Å from the bilayer midplane indicating that it is likely to be surface bound or unbound. A coexistence of inserted and surface bound A*β*25-35 states has been observed for the weakly anionic 97:3 DMPC/DMPS bilayer and 3 mol% of A*β*25-35^41^. Those findings are qualitatively consistent with our results, which were obtained at 4 mol% of peptide^34^. As a result of deeper penetration into the bilayer all amino acids form stronger interactions with lipids. However, it is the hydrophobic C-terminus amino acids Ala30 and Leu34 and cationic Lys28 that demonstrate the largest gain in binding interactions. Therefore, in agreement with the aggregation mechanisms proposed by Bokvist *et al*., a combination of hydrophobic effect and electrostatic interactions drives an increase in binding affinity of A*β*25-35 oligomers^26^.

Second, our findings indicate that A*β*25-35 aggregation leads to stronger disruption in the bilayer structure. This conclusion follows from a pronounced lipid density void, which emerges in the DMPC bilayer when the inserted A*β* dimer displaces lipids. Indeed, aggregation increases the extent of bilayer thinning Δ*D* more than four-fold compared to monomeric species. As a result, A*β*25-35 dimer reduces the thickness of the DMPC bilayer almost 40%, from 40 Å^42^ to ≈24 Å. Simultaneously, a three-fold drop in lipid surface number density is observed within the A*β*25-35 dimer binding footprint, which is far more dramatic than that caused by A*β*25-35 monomers^34^. We point out that the thinning observed in our simulations is not related to hydrophobic mismatch occurring between the thickness of hydrophobic lipid core and the length of protein hydrophobic transmembrane region. Instead, it is created by A*β*25-35 peptides displacing lipids from the volume of bilayer and effectively plugging the lipid void. We believe this was the reason why X-ray scattering experiments did not detect a decrease in bilayer thickness upon binding of A*β*25-35 at various concentrations^43^. Our analysis of carbon-deuterium order parameter and lipid tilt angles revealed that disordering in DMPC fatty acid tails increases with aggregation. The same trend in tilt angles has been seen experimentally for POPC/DMPS bilayer as a function of peptide concentration^43^.

Our third result pertains to the structure of the A*β*25-35 dimer. The free energy landscape in Fig. 5 has revealed that A*β*25-35 dimers inserted into the DMPC bilayer represent the most thermodynamically stable state **ID** gathering more than half of A*β*25-35 peptides. Structurally, this state is homogeneous as an overwhelming fraction of dimers can be grouped into a single dominant cluster *ID*1 shown in Fig. 5. The centroid of this cluster exhibits a dimer, which utilizes a parallel out-of-registry aggregation interface composed of the sequence regions Gly33-Met35 in the left peptide and Gly29-Ile31 in the right peptide (the inset to Fig. 6). The interface draws its stability from three stable hydrophobic contacts (Gly29-Gly33, Ala30-Leu34, Ile31-Met35) and backbone hydrogen bonds. Thus, the structure of A*β*25-35 dimer uses an unusual helix head-to-tail tandem of peptides, which differs from A*β* fibril conformations rich in *β*-structure^44^ or the structures of C99 homodimers in the lipid bilayers. The NMR-resolved conformations and molecular dynamics simulations of the latter exhibit two helices associated via their sides, which depending on the environment may or may not involve Gly-Gly heptad interactions^45–47^. However, similar to C99 dimerization, A*β*25-35 dimer incorporates peptides in monomer-like conformations. Our analysis in Supplementary Information (Fig. S6) confirms that the distributions of helical structure and intrapeptide interactions are similar between **ID** dimers, inserted **IM** monomers, and A*β*25-35 inserted monomers **I** sampled in our previous REST simulations^34^. Thus, A*β*25-35 aggregates by recruiting monomeric peptides without radical reorganization in their structure. This outcome is in line with recent X-ray scattering experiments, which showed A*β*25-35 peptide to retain its monomeric *α*-helical structures up to the concentration of 3 mol%, which is almost identical to that in our simulations (4 mol%)^43^. It is likely that the helix head-to-tail topology of A*β*25-35 dimer is relevant for aggregation of antimicrobial peptides^48^. Similar to A*β*25-35 these short peptides typically adopt an amphipathic helical structure upon binding to anionic bacterial membranes. It is conceivable that their aggregation proceeds via head-to-tail assembly of monomeric units as shown in inset to Fig. 6.

An intriguing feature of the **ID** dimer is that it readily affords recruitment of new peptides in the same monomer-like conformations by adding them either to the left or right available aggregation interfaces (the inset to Fig. 6). Therefore, the A*β*25-35 dimers identified in our simulations may represent building blocks, which by replicating their structure may grow into larger annular aggregates detected by recent AFM studies^36^. Since A*β*25-35 dimers efficiently displace lipids, one can expect that such annular structures with a diameter of about 20 nm^36^ and constructed of dozens of A*β*25-35 dimeric units may form stable pores for ion permeation as shown in Fig. 6. Such hypothetical pore may be constructed of several concentric rings of A*β*25-35 peptides. Note that A*β*25-35 dimers utilize not only helix head-to-tail **ID** topology, but also helix side-by-side antiparallel aggregation interface in **SBD**. Taking into account the amphiphilic character of A*β*25-35 dimer, one can envision that in the inner ring the polar N-termini of the peptides are pointed toward the center of the pore, whereas the hydrophobic C-terminal helices are bound to the helices of the peptides from the two outer rings in antiparallel fashion as in **SBD**. Therefore, the proposed structure of a pore features a polar lining made of N-terminal regions of A*β*25-35, which should stabilize a pore through favorable hydration.

Finally, REST sampling enabled us to construct in Fig. 5 the free energy landscape *G*(*Z*_*d*_, *R*) governing A*β* binding and aggregation. It shows that the peptides populate three states or basins. The most thermodynamically stable state **ID** populated by inserted dimers and discussed above is supplemented by two metastable states - inserted **IM** monomers and **SBD** dimers bound to the surface of the DMPC bilayer. Because the states **IM** and **SBD** are separated from **ID** by the free energy gaps of ~1 kcal/mol and the free energy barriers surrounding these states are modest ($\lesssim$4 kcal/mol), A*β*25-35 peptides may interconvert between these states while still predominantly sampling **ID**. Interestingly, Fig. 5 does not implicate an existence of stable A*β*25-35 monomers bound to the DMPC bilayer surface suggesting that such species rapidly aggregate forming a state **SBD**. Absence of monomeric surface bound species also demonstrates that DMPC lipid bilayer acts as an aggregation catalyst. Because the free energy landscape *G*(*Z*_*d*_, *R*) does not feature a low free energy path between **IM** and **SBD**, we expect the following scheme to depict A*β*25-35 kinetics:

1 $${\bf{S}}{\bf{B}}{\bf{D}}\overleftarrow{{\boldsymbol{\to }}}{\bf{I}}{\bf{D}}\overrightarrow{{\boldsymbol{\leftarrow }}}{\bf{I}}{\bf{M}}.$$

Eq. (1) suggests the aggregation pathway at low A*β*25-35 concentrations. The peptides bind to the DMPC bilayer as monomers^28^ and rapidly aggregate into **SBD** dimers on the bilayer surface. Next, **SBD** A*β*25-35 dimers penetrate the DMPC bilayer becoming inserted dimers **ID**. While the peptides overwhelmingly retain **ID** dimeric form, they may transiently dissociate into inserted monomers **IM** or move to the bilayer surface to sample **SBD** dimers. Direct insertion of surface bound monomers into the bilayer is disfavored, because according to scenario (1) these species are redirected toward forming surface bound dimers. It is important to point out that A*β*25-35 aggregation does not involve radical restructuring of monomeric peptides, which are integrated largely intact into dimers.

The aggregation scenario depicted above is broadly consistent with the three-stage aggregation pathway proposed by Cuco *et al*., which includes adsorption, nucleation, and penetration^49^. In that hypothetical pathway, nucleation of adsorbed peptides promotes their penetration into the bilayer in agreement with our prediction that aggregation drastically increases the population of inserted A*β*25-35 species in Fig. 3b. The aggregation scenario suggested by our simulations should only be valid at low peptide concentrations, which favor *α*-helical A*β*25-35 structures^43^, whereas higher peptide concentrations lead to *β*-barrel aggregates^37,38^. The effects of bilayer composition and post-translational peptide modifications on A*β*25-35 aggregation and Ca^2+^ permeation are discussed in Supplementary Information. In addition, the free energy landscape in Fig. 5 may underestimate the slope toward aggregated surface bound species due to neglect of bilayer curvature and associated surface tension effects^43^. However, these neglected factors are expected to disfavor the formation of surface bound monomers even further than predicted by our findings.

In summary, our all-atom explicit solvent REST simulations provide the first, to our knowledge, description of A*β* peptide *de novo* aggregation mediated by a lipid bilayer. The microscopic details concerning the mechanism of A*β* binding, aggregation, and insertion into the bilayer together with the disruption in the bilayer structure are expected to advance our understanding of the molecular causes of A*β* cytotoxicity.

## Models and Methods

### Simulation system

Our simulation system consisted of four A*β*25-35 peptides, dimyristoyl phosphatidylcholine (DMPC) bilayer, water, and counterions (Fig. 1). The all-atom CHARMM22 force field with CMAP corrections^50^ and the all-atom CHARMM36 force field^51^ were used to represent peptides and lipids, respectively. For water we used a modified TIP3P model^52^. Neutral acetylated and amidated groups were used to cap the peptides. A pair of A*β*25-35 monomers was placed on each side of the bilayer, each leaflet of which was formed by 50 DMPC molecules. The solvent was comprised of 4344 water molecules, and four chloride counterions were added. The total number of atoms was 25,480, and the initial unit cell dimensions were approximately 56 Å × 56 Å × 78 Å. The design of our simulation system affords probing simultaneous binding of two A*β*25-35 dimers and reduces the possibility of the development of bilayer curvature^53^.

### Replica exchange simulations

To sample the conformational ensemble we used isobaric-isothermal replica exchange with solute tempering (REST) molecular dynamics simulations^54^. Because REST formalism is documented elsewhere^54,55^, we provide here its brief outline. In all, the temperatures of $R=8$ replicas were distributed geometrically from *T*_0_ = 330 K to *T*_*R*−1_ = 430 K. An exchange between replicas *r* and *r* + 1 occurs with the probability $\omega =\,{\min }\,[1,{e}^{-{\rm{\Delta }}}]$, where ${\rm{\Delta }}={\beta }_{r}({H}_{r}({X}_{r+1})-{H}_{r}({X}_{r}))+{\beta }_{r+1}({H}_{r+1}({X}_{r})-{H}_{r+1}({X}_{r+1}))$, $\beta ={(RT)}^{-1/2}$, *H* is the enthalpy, and *X* defines system coordinates. Solvent-solvent and solute-solvent interactions in replica *r* with the temperature *T*_*r*_ were scaled by the factors *T*_*r*_/*T*_0_ and (*T*_*r*_/*T*_0_)^1/2^, respectively. This scaling excludes solvent-solvent energy contributions from $\omega$ and reduces the number of replicas without affecting the temperature range or exchange rates. A*β* peptides and ions were treated as solute, whereas lipids and water were considered as solvent. Replica exchanges were attempted every 2 ps succeeding with the probability of 0.28.

To perform REST simulations we used the program NAMD^56^ with REST implementation^57^ and in-house scripts for managing replicas and exchanges. Periodic boundary conditions were utilized. Covalent bonds associated with hydrogen atoms were constrained by the SHAKE algorithm. Electrostatic interactions were computed using Ewald summations, and van der Waals interactions were smoothly switched off from 8 to 12 Å. Underdamped Langevin dynamics with a damping coefficient *γ* = 5 ps^−1^ was used to control temperature, and the Nose-Hoover Langevin piston method with piston period and decay of 200 and 100 fs, respectively, was used to set pressure at 1 atm. The *x* and *y* dimensions of the system were coupled, and the *z* dimension along the bilayer normal fluctuated independently. An integration timestep of 1 fs was used. To preserve bilayer integrity at high REST temperatures we applied harmonic restraints to the centers of mass of phosphorus atoms in each leaflet as described^58^. Another set of restraints at *z* periodic boundaries prevented aggregation of A*β*25-35 dimers^34^. Impact of these restraints is negligible and has been discussed previously^34^.

To prepare starting structures for REST simulations we used the conformations of A*β*25-35 peptides, which were inserted or surface bound to the DMPC bilayer^34^. These initial structures, in which the peptides were temporarily constrained along the bilayer normal, were equilibrated at 440 K for 30 ns. The procedure generated diverse structures, in which peptides form random interpeptide interactions and half of them were surface bound to the bilayer and half were inserted. These starting conformations were used to initialize six REST trajectories, which produced 2.88 *μs* of sampling (360 *ns* per replica). By monitoring REST convergence (see Supplementary Information), we excluded 40 ns of sampling per replica in each trajectory as non-equilibrated. Thus, the total equilibrium sampling is reduced to 0.96 *μs* or 1.92 *μs* per dimer.

### Computation of structural probes

To facilitate analysis we divided A*β*25-35 peptide into the polar N-terminal R3 and the hydrophobic C-terminal R4 regions (Fig. 1a). A DMPC lipid consists of five structural groups as shown in Fig. 1b. These include choline (L1) and phosphate (L2) groups, glycerol backbone (L3) and two fatty acid tails (L4 and L5). Intra- and intermolecular interactions were detected using the positions of centers of mass of amino acid side chains and lipid structural groups. A contact between them occurs if the distance between their centers of mass is less than 6.5 Å. A*β*25-35 dimer is formed if at least one interpeptide contact is established. A*β*25-35 secondary structure was assigned using the program STRIDE^59^, and *α*-, 3_10_-, or *π* helices were combined into a helical state.

To probe A*β*25-35 penetration into the bilayer, we defined the probability *P*(*z*; *i*) for an amino acid *i* to occur at a distance *z* from the bilayer midplane. An amino acid is considered inserted if it resides below the average position of the center of mass of phosphorus atoms *z*_*P*_. Similarly, an amino acid is bilayer surface bound if it occurs within the lipid headgroup region (*z*_*p*_ < *z* < *z*_*P*_ + 6.5 Å). The number density *n*_*l*_(*r*, *z*) of DMPC heavy atoms with respect to the distance *r* to the A*β*25-35 peptide center of mass and the distance *z* to the bilayer midplane mapped the impact of peptides on the bilayer structure. Using *n*_*l*_(*r*, *z*) we defined the bilayer boundary *z*_*b*_(*r*) and thickness *D* as described^34^. To examine lipid disordering, carbon-deuterium order parameter *S*_*CD*_ was computed for carbons 2 through 14 in the *sn* − 2 fatty acid tails. We also computed the tilt angle *γ* measured between the bilayer normal and the vector connecting the first and last carbons in *sn* − 2 fatty acid tail. DMPC lipids were divided into distant occurring at the distance *r* > 20 Å from the A*β*25-35 peptide center of mass and proximal occurring within the center of binding footprint ($r < 4\AA  < {\textstyle \tfrac{1}{2}}\langle {R}_{g}\rangle =4.7\AA$, where 〈*R*_*g*_〉 is A*β*25-35 radius of gyration). All structural probes are reported as thermodynamic averages computed for the wild-type replica at *T*_0_ = 330 K and denoted with angular brackets 〈..〉. Slightly elevated temperature facilitated conformational sampling and comparisons with our previous A*β*25-35 monomer simulations^34^.

### Conformational clustering

Clustering of peptide conformations was performed using the method of Daura *et al*.^60^ implemented in VMD^61^. To this end, we first computed the free energy landscape of A*β*25-35 dimers *G*(*Z*_*d*_, *R*) as a function of the z-position of the center of mass of two peptides *Z*_*d*_ and the distance between their centers of mass *R*. Clustering was applied separately to the structures populating each of the three *G*(*Z*_*d*_, *R*) basins (see Table 1 and Fig. 5). For each pair of A*β*25-35 dimers from **ID** or **SBD** aggregated states, we computed RMSD between backbone C*α* atoms after dimer alignment and permuting the identities of peptides. In the dissociated basin **IM**, RMSD values were computed between peptide monomers. Dimer or monomer clusters were defined by the RMSD cutoff value *R*_0_. To select *R*_0_, we examined clustering at various cutoffs selecting only populated clusters, each of which must represent at least 5% of basin structures and together they must capture at least 50% of basin structures. Then *R*_0_ is the largest RMSD cutoff that occurs prior to a major reorganization of clusters such as when two smaller distinct clusters merge into one larger cluster. The clusters were assumed distinct if they exhibit different aggregation interfaces or peptide conformations. Structural properties of a cluster are reported as averages over the conformations populating a cluster.

## Supplementary information

Supplementary Information

## Data Availability

The datasets generated and analysed during the current study are available from the corresponding author on reasonable request.

## References

[1] Petkova AT, Yau W-M, Tycko R (2006). Experimental constraints on quaternary structure in Alzheimer’s *β*-amyloid fibrils. Biochemistry.

[2] Serpell LC (2000). Alzheimer’s amyloid fibrils: Structure and assembly. Biochim Biophys. Acta.

[3] Pike C, Burdick D, Walencewicz A, Glabe C, Cotman C (1993). Neurodegeneration induced by *β*-amyloid peptides *in vitro*: The role of peptide assembly state. J. Neurosci..

[4] Williams TL, Serpell LC (2011). Membrane and surface interactions of Alzheimer’s A*β* peptide - insights into the mechanism of cytotoxicity. FEBS J..

[5] Sergeant N (2003). Truncated beta-amyloid peptide species in pre-clinical Alzheimers disease as new targets for the vaccination approach. J. Neurochem..

[6] Christensen R, Marcussen A, Wortwein G, Knudsen G, Aznar S (2008). A*β*(1-42) injection causes memory impairment, lowered cortical and serum bdnf levels, and decreased hippocampal 5-ht(2a) levels. Exp. Neurol..

[7] Haass C, Selkoe DJ (2007). Soluble protein oligomers in neurodegeneration: Lessons from the Alzheimers amyloid *β*-peptide. Nature Rev. Mol. Cell. Biol..

[8] Shankar GM (2008). Amyloid-*β* protein dimers isolated directly from Alzheimers brains impair synaptic plasticity and memory. Nature Medicine.

[9] Hardy J, Higgins GA (1992). Alzheimer’s disease: The amyloid cascade hypothesis. Science.

[10] Maddalena A (2004). Cerebrospinal fluid profile of amyloid *β* peptides in patients with Alzheimer’s disease determined by protein biochip technology. Neurodegener. Dis..

[11] Millucci L, Ghezzi L, Bernardini G, Santucci A (2010). Conformations and biological activities of amyloid *β* peptide 25-35. Curr Protein Pept Sci.

[12] Kaneko, I., Yamada, N., Usui, Y. & Oda, T. Possible involvement of *β*-amyloids racemized at ser residue in Alzheimer’s disease. In *Alzheimer*’*s Disease: Biology*, *Diagnose and Therapeutics*, 519–528, Chichester (John Wiley & Sons, 1997).

[13] Pike CJ (1995). Structure-activity analyses of beta-amyloid peptides: Contributions of the beta 25-35 region to aggregation and neurotoxicity. J. Neurochem..

[14] Sato K (1995). Correlation among secondary structure, amyloid precursor protein accumulation, and neurotoxicity of amyloid *β*(25-35) peptide as analyzed by single alanine substitution. J. Biochem..

[15] Clementi ME (2005). A*β*(31-35) and A*β*(25-35) fragments of amyloid beta-protein induce cellular death through apoptotic signals: Role of the redox state of methionine-35. FEBS.

[16] Tsai H-HG (2014). Location and conformation of amyloid *β*(25-35) peptide and its sequence-shuffled peptides within membranes: Implications for aggregation and toxicity in PC12 cells. Chem. Med. Chem..

[17] Song Y, Li P, Liu L, Bortolini C, Dong M (2018). Nanostructural differentiation and toxicity of amyloid-*β*25-35 aggregates ensue from distinct secondary conformation. Sci. Rep..

[18] Tepanichev MY, Moiseeva YV, Lazareva NA, Gulyaeva NV (2005). Studies of the effects of fragment (25-35) of beta-amyloid peptide on the behavior of rats in a radial maze. Neurosci. Behav. Physiol..

[19] Limon ID (2009). Amyloid-beta(25-35) impairs memory and increases NO in the temporal cortex of rats. Neurosci. Res..

[20] Frozza F (2009). A comparative study of *β*-amyloid peptides a*β*1-42 and a*β*25-35 toxicity in organotypic hippocampal slice cultures. Neurochem. Res..

[21] Misiti F (2005). A*β*(31-35) peptide induce apoptosis in pc 12 cells: contrast with a*β*(25-35) peptide and examination of underlying mechanisms. Neurochem. Int..

[22] Tsai HHG, Lee JB, Tseng SS, Pan XA, Shih YC (2010). Folding and membrane insertion of amyloid-beta (25-35) peptide and its mutants: Implications for aggregation and neurotoxicity. Proteins Struct. Funct. Bioinform..

[23] Larini L, Shea J-E (2012). Role of *β*-hairpin formation in aggregation: The self-assembly of the amyloid-*β*(25-35) peptide. Biophys. J..

[24] Terzi E, Hölzemann G, Seelig J (1997). Interaction and Alzheimer’s *β*-amyloid peptide(1-40) with lipid membranes. Biochemistry.

[25] Yip CM, McLaurin J (2001). Amyloid-*β* peptide assembly: a critical step in fibrillogenesis and membrane disruption. Biophys. J..

[26] Bokvist M, Lindström F, Watts A, Gröbner G (2004). Two types of Alzheimer’s *β*-amyloid (1-40) peptide membrane interactions: Aggregation preventing transmembrane anchoring versus accelerated surface fibril formation. J. Mol. Biol..

[27] Quist A (2005). Amyloid ion channels: A common structural link for protein-misfolding disease. Proc. Natl. Acad. Sci. USA.

[28] Nag S, Chen J, Irudayaraj J, Maiti S (2010). Measurement of the attachment and assembly of small amyloid-*β* oligomers on live cell membranes at physiological concentrations using single-molecule tools. Biophys. J..

[29] Ambroggio EE (2005). Surface behavior and lipid interaction of Alzheimer’s *β*-amyloid peptide 1-42: A membrane-disrupting peptide. Biophys. J..

[30] Terzi E, Holzemann G, Seelig J (1994). Alzheimer *β*-amyloid peptide 25-35: Electrostatic interactions with phospholipid membranes. Biochemistry.

[31] Dante S, Hauss T, Dencher NA (2003). Insertion of externally administered amyloid *β* peptide 25-35 and perturbation of lipid bilayers. Biochemistry.

[32] DÉrrico G (2008). Interaction between Alzheimer’s a*β*(25-35) peptide and phospholipid bilayers: The role of cholesterol. Biochimica et Biophysica Acta.

[33] Mason RP, Estermyer JD, Kelly JF, Mason PE (1996). Alzheimer’s disease amyloid *β* peptide 25-35 is localized in the membrane hydrocarbon core: X-ray diffraction analysis. Biochem. Biophys. Res. Commun..

[34] Smith AK, Klimov DK (2018). Binding of cytotoxic a*β*25-35 peptide to the dmpc lipid bilayer. J. Chem. Inform. Model..

[35] Krstic D, Knuesel I (2013). Deciphering the mechanism underlying late-onset Alzheimer disease. Nat Rev Neurol.

[36] Saponetti MS (2014). Aggregation of a*β*(25-35) on DOPC and DOPC/DHA bilayers: An atomic force microscopy study. Plos One.

[37] Kandel N, Zheng T, Huo Q, Tatulian SA (2017). Membrane binding and pore formation by a cytotoxic fragment of amyloid *β* peptide. J. Physc. Chem. B.

[38] Kandel N, Matos JO, Tatulian SA (2019). Structure of amyloid *β*25–35 in lipid environment and cholesterol dependent membrane pore formation. Sci. Reports.

[39] Shai Y (2002). Mode of action of membrane active antimicrobial peptides. Biopolymers.

[40] Dante S, Hauss T, Dencher NA (2002). *β*-amyloid 25 to 35 is intercalated in anionic and zwitterionic lipid membranes to different extents. Biophys. J..

[41] Dies H, Toppozini L, Rheinstadter MC (2014). The interaction between amyloid-*β* peptides and anionic lipid membranes containing cholesterol and melatonin. PLoS One.

[42] Lockhart C, Klimov DK (2016). The Alzheimer’s disease A*β* peptide binds to the anionic DMPS lipid bilayer. BBA Biomembranes.

[43] Tang J (2016). Amyloid-*β*25–35 peptides aggregate into cross-*β* sheets in unsaturated anionic lipid membranes at high peptide concentrations. Soft Matter.

[44] Paravastua AK, Leapman RD, Yaua W-M, Tycko R (2008). Molecular structural basis for polymorphism in Alzheimers *β*-amyloid fibrils. Proc. Natl. Acad. Sci. USA.

[45] Nadezhdin KD, Bocharova OV, Bocharov EV, Arseniev AS (2012). Dimeric structure of transmembrane domain of amyloid precursor protein in micellar environment. FEBS Lett.

[46] Wang H (2011). Molecular determinants and thermodynamics of the amyloid precursor protein transmembrane domain implicated in Alzheimer’s disease. J. Mol. Biol..

[47] Dominguez L, Foster L, Straub JE, Thirumalai D (2016). Impact of membrane lipid composition on the structure and stability of the transmembrane domain of amyloid precursor protein. Proc. Natl. Acad. Sci. USA.

[48] Dathe M, Wieprecht T (1999). Structural features of helical antimicrobial peptides: their potential to modulate activity on model membranes and biological cells. Biochimica et Biophysica Acta.

[49] Cuco A, Serro AP, Farinha JP, Saramago B, da Silva AG (2016). Interaction of the Alzheimer a*β*(25–35) peptide segment with model membranes. Coll. Surf. B Biointerfaces.

[50] Buck M, Bouguet-Bonnet S, Pastor RW, MacKerell AD (2006). Importance of the CMAP correction to the CHARMM22 protein force field: Dynamics of hen lysozyme. Biophys. J..

[51] Klauda JB (2010). Update of the CHARMM all-atom additive force field for lipids: validation on six lipid types. J. Phys. Chem. B.

[52] MacKerell AD (1998). All-atom empirical potential for molecular modeling and dynamics studies of proteins. J. Phys. Chem. B.

[53] Park S, Beaven AH, Klauda JB, Im W (2015). How tolerant are membrane simulations with mismatch in area per lipid between leaflets?. J. Chem. Theor. Comput..

[54] Wang L, Friesner RA, Berne BJ (2011). Replica exchange with solute scaling: A more efficient version of replica exchange with solute tempering (REST2). J. Phys. Chem. B.

[55] Smith AK, Lockhart C, Klimov DK (2016). Does replica exchange with solute tempering efficiently sample A*β* peptide conformational ensembles?. J. Chem. Theor. Comp..

[56] Kalé L (1999). NAMD2: Greater scalability for parallel molecular dynamics. J. Comput. Phys..

[57] Jo S, Jiang W (2015). A generic implementation of replica exchange with solute tempering (REST2) algorithm in NAMD for complex biophysical simulations. Comput. Phys. Commun..

[58] Lockhart C, Klimov DK (2014). Alzheimer’s A*β*10-40 peptide binds and penetrates DMPC bilayer: An isobaric-isothermal replica exchange molecular dynamics study. J. Phys. Chem. B.

[59] Frishman D, Argos P (1995). Knowledge-based protein secondary structure assignment. Proteins Struct. Funct. Gen..

[60] Daura X (1999). Peptide folding: When simulation meets experiment. Angew. Chem. Int. Ed..

[61] Humphrey W, Dalke A, Schulten K (1996). VMD – Visual Molecular Dynamics. J. Mol. Graph.

