# Supplementary material for: De novo aggregation of Alzheimer’s Aβ25-35 peptides in a lipid bilayer
Source: Sci Rep. 2019 May 9;9:7161. doi: 10.1038/s41598-019-43685-7 (PMC6509337; doi:10.1038/s41598-019-43685-7)
Supplement: Supplementary file 1 — Supplementary Information [file 41598_2019_43685_MOESM1_ESM.pdf]

De novo aggregation of Alzheimer's Aβ25-35 peptides in a lipid bilayer

Amy K. Smith and Dmitri K. Klimov
School of Systems Biology, George Mason University, Manassas, VA 20110
E-mail: dklimov@gmu.edu

Supplementary Information

Assessment of REST performance: We used replica exchange with solute tempering (REST) molecular dynamics simulations to sample the conformational ensemble of Aβ25-35 dimers binding to the DMPC bilayer. REST simulations utilized R=8 replicas distributed in the temperature range from 330K to 430K. One of the prerequisites of efficient conformational sampling in REST is the implementation of replica random walk over temperatures. A representative replica walk is visualized in Fig. S1. An emerging random color mosaic suggests that no replica is trapped at any temperature implicating efficient replica mixing across temperatures.

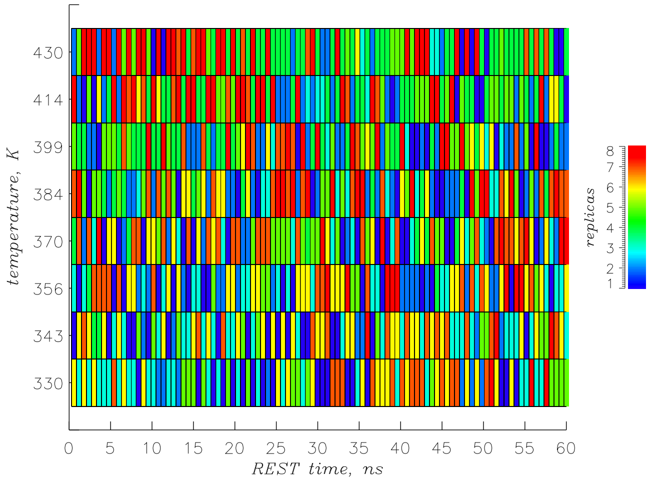

Figure S1. Random walk of replicas over temperatures in a representative REST trajectory. The distribution of replicas over temperatures at the trajectory start is color-coded according to the scale at the right.

Han and Hansmann [1] have proposed a quantitative measure of replica mixing defined as

$$m(T) = 1 - \frac{\sqrt{\sum_{r=0}^{R-1} t_r^2}}{\sum_{r=0}^{R-1} t_r}, \qquad (1)$$

where T is the REST temperature and t_r is the total number of REST iterations spent at T by replica r. If the total number of replicas is R=8, then the optimum theoretical value of $m(T)$ for any temperature is $1 - 1/R^{1/2} = 0.65$. In Fig. S2 we display the measure $m(T)$ after averaging over all REST trajectories. Although there are some deviations near the ends of temperature range due to boundary effects, $m(T)$ approaches the theoretical value at most REST temperatures. Thus, this figure suggests nearly ideal replica mixing.

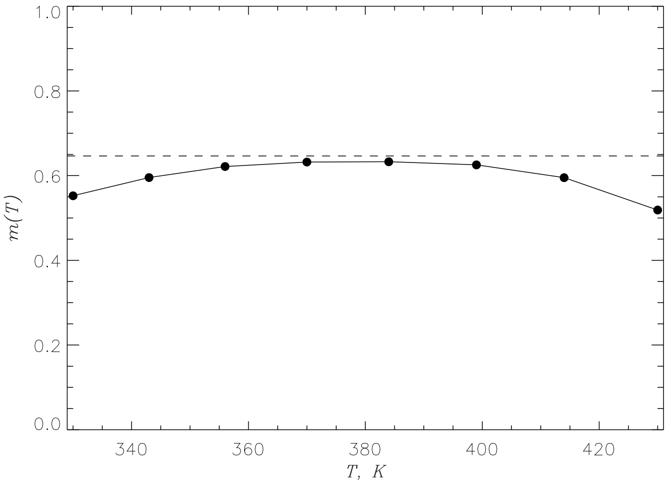

Figure S2. The mixing measure $m(T)$ as a function of the REST temperature T. Dashed line indicates the optimum theoretical value of 0.65.

Conformational sampling in REST simulations: Our REST trajectories have been initiated with an equal mix of peptides that are surface bound or inserted into the DMPC bilayer. In all, we have produced six REST trajectories, in which each of eight replicas was simulated for 60 ns. To monitor equilibration, we first considered the probability distributions $P(Z_m)$ of the z-position of Aβ25-35 peptide center of mass Z_m along the DMPC bilayer normal. These distributions were computed at 330K over the six batches of 10 ns each using all six trajectories. Six corresponding distributions $P(Z_m)$ are presented in Fig. S3a. The first four distributions collected over first 40 ns of sampling demonstrate shifting probabilities of surface bound and inserted states reflecting the process of equilibration of binding of Aβ25-35 dimers to the bilayer. However, the two distributions collected over the last 20 ns of sampling are nearly identical suggesting settling of the system in equilibrium state. The distance R between the centers of mass of Aβ25-35 peptides forming a dimer in a leaflet can be used as a second measure of aggregation equilibration. To this end, we plot in Fig. S3b the distance R, which is averaged over six REST trajectories and pairs of dimers, as a function of REST time. If interactions between Aβ25-35 peptides are at equilibrium, R should reach a plateu as a function of REST simulation time. Fig. S3b shows that this is indeed the case after approximately 40 ns of sampling. Thus, we determine that the equilibration time in our REST simulations of Aβ25-35 dimers is $\tau_{eq}\approx$40 ns.

(a)

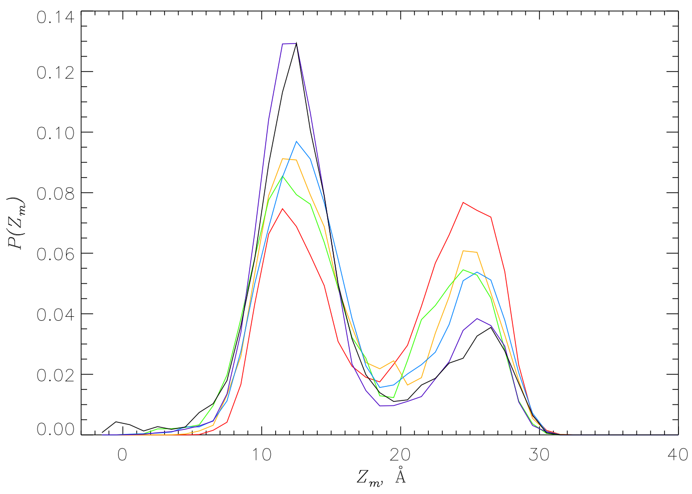

(b)

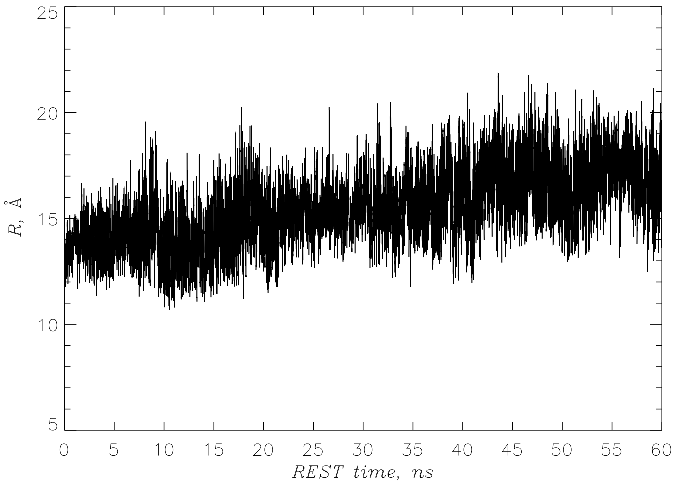

Figure S3. (a) Probability distributions $P(Z_m)$ of the position of Aβ25-35 peptide center of mass Z_m along the DMPC bilayer normal computed over the batches of 10 ns sampling. The batches are color coded as red (0-10 ns), yellow (10-20 ns), green (20-30 ns), blue (30-40 ns), purple (40-50 ns), and black (50-60 ns). (b) The distance R between the centers of mass of Aβ25-35 peptides in a dimer as a function of REST simulation time.

Aβ25-35 secondary structure: Table S1 compares secondary structure in Aβ25-35 monomers [2] and dimers by listing the average probabilities to observe helical $<H>$, β–turn $<T>$, and random coil $<RC>$ conformations. It reveals a moderate increase in the helical fraction $<H>$ coupled with a simultaneous decrease in the turn content $<T>$. Similar but more pronounced changes are observed in the R4 C-terminus, which features a stable helical structure (>0.50).

Table S1 Secondary structure propensities in Aβ25-35[a].

System	Quantity	entire peptide	R4
**Aβ monomer**[b]	$\langle \boldsymbol{H} \rangle$	0.31±0.03	0.39±0.02
	$\langle \boldsymbol{T} \rangle$	0.36±0.02	0.34±0.02
	$\langle \boldsymbol{RC} \rangle$	0.33±0.02	0.27±0.02
**Aβ dimer**	$\langle \boldsymbol{H} \rangle$	0.39±0.03	0.54±0.03
	$\langle \boldsymbol{T} \rangle$	0.27±0.03	0.23±0.03
	$\langle \boldsymbol{RC} \rangle$	0.33±0.02	0.23±0.02

[a] Highlighted cells mark stable secondary structure.
[b]Data from [2].

Aβ25-35 intrapeptide interactions: Effect of aggregation on intrapeptide interactions was assessed via the difference contact map $\langle \Delta C(i,j)\rangle = \langle C(i,j)\rangle - \langle C(i,j)\rangle_M$, where $\langle C(i,j)\rangle$ and $\langle C(i,j)\rangle_M$ are the dimer and monomer [2] contact maps reporting the formation of contacts between amino acids *i* and *j*. The contacts most affected by aggregation ($|\langle \Delta C(i,j)\rangle| \geq 0.1$) are shown in Table S2. These interactions reflect stabilization of helical structure in Aβ25-35 dimers. In fact, two helix contacts (Gly29-Leu32, Gly29-Gly33) became particularly stable as their probability of formation reaches 0.74 and 0.70, respectively. However, because only three contacts out of 55 topologically possible are noticeably affected by aggregation and two of them (Gly29-Leu32, Gly29-Gly33) are already stable in Aβ25-35 monomers ($\langle C(i,j)\rangle_M > 0.4$) [2], we surmise that aggregation does not cause a radical change in Aβ25-35 tertiary structure.

Table S2 List of intrapeptide contacts affected by aggregation.

Rank	*i*	*j*	$\langle \Delta C(i,j)\rangle$
1	29	33	0.22
2	29	32	0.14
3	28	33	0.12

Aβ25-35 interactions with the DMPC bilayer: Differences in the binding mechanism between Aβ25-35 dimers and monomers were explored using the contact map $\langle C_l(i,k)\rangle$, which reports the formation of contacts between amino acids *i* and lipid groups *k* . Fig. S4 shows the contact maps for Aβ25-35 dimers and monomers as well as the difference in the number of contacts with lipids per amino acid $\langle \Delta C_l(i)\rangle = \langle C_l(i)\rangle - \langle C_l(i)\rangle_M$, where $\langle C_l(i)\rangle$ is the number of contacts formed by amino acid *i* with all lipid groups and subscript *M* refers to monomer [2]. The figure shows that peptide aggregation enhances interactions of all Aβ25-35 amino acids with the DMPC bilayer. Detailed analysis of amino acid - lipid interactions is given in the main text.

(a)

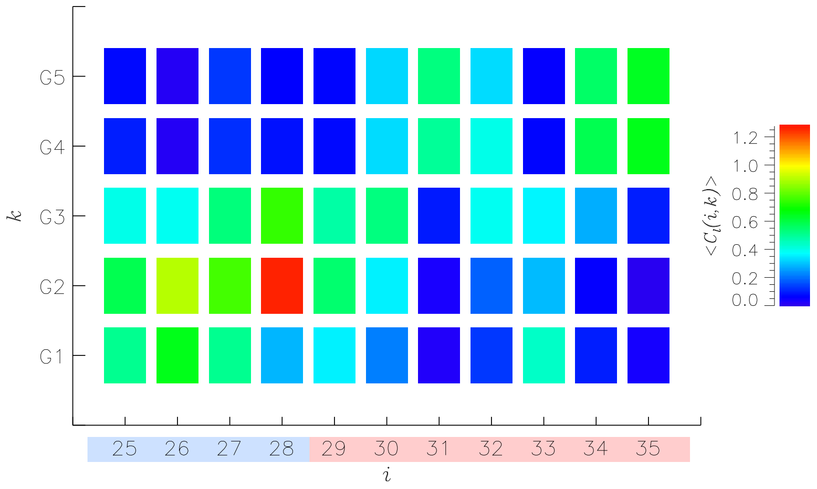
G5
G4
G3
G2
G1
k
25 26 27 28 29 30 31 32 33 34 35
i
1.2
1.0
0.8
0.6
0.4
0.2
0.0
<C_l(i,k)>

(b)

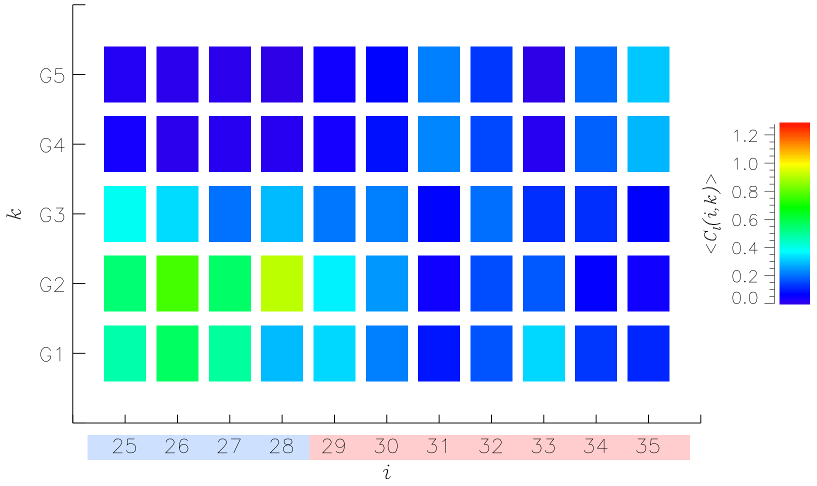
G5
G4
G3
G2
G1
k
25 26 27 28 29 30 31 32 33 34 35
i
1.2
1.0
0.8
0.6
0.4
0.2
0.0
<C_l(i,k)>

(c)

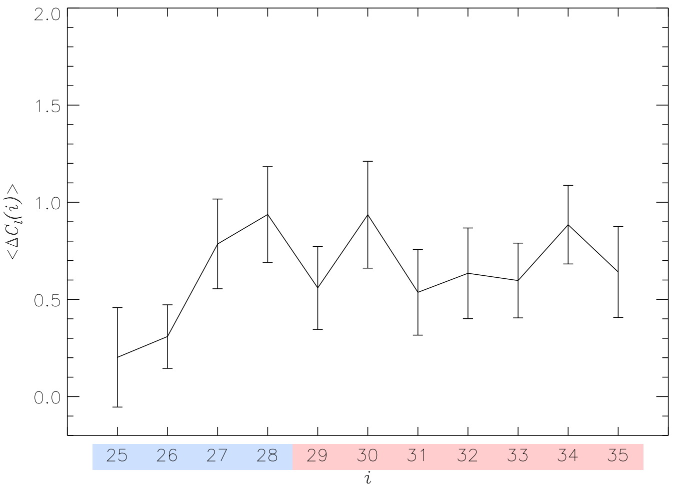
2.0
1.5
1.0
0.5
0.0
<ΔC_l(i)>
25 26 27 28 29 30 31 32 33 34 35
i

Fig. S4 The contact maps $<C_l(i,k)>$ report the formation of contacts between amino acids i and lipid structural groups k . Panel (a) and (b) describe the interactions of Aβ25-35 dimers and monomers with the DMPC bilayer, respectively. (c) The difference $<\Delta C_l(i)>$ identifies changes in the number of contacts with the bilayer per amino acid caused by the aggregation. Sequence regions R3-R4 are colored according to Fig. 1a.

Impact of Aβ25-35 dimer on the DMPC bilayer: To evaluate conformational changes in the DMPC fatty acid tails induced by Aβ25-35 binding, we computed the lipid carbon-deuterium order parameter $-<S_{CD}(i)>$ for each carbon i in *sn-2* chains. The corresponding plots shown in Fig. S5a indicate that lipids proximal to Aβ25-35 peptide are structurally disordered. Indeed, it follows from the inset to Fig. S5a that the average difference in $-<S_{CD}(i)>$ between proximal and distant lipids is $-<\Delta S_{CD}>=0.054\pm0.009$ for Aβ25-35 dimers and 0.037±0.008 for the monomers. Fig. S5b presents the probability distribution $P(\gamma)$ of tilt angles γ for distant and proximal lipids. It follows that Aβ25-35 dimers increase the average tilt by $<\Delta\gamma>=7\pm1°$, whereas the corresponding change in γ induced by Aβ25-35 monomers [2] is smaller (5±1°). These findings indicate that Aβ25-35 dimers disorder proximal lipids to a larger extent than Aβ25-35 monomers.

(a)

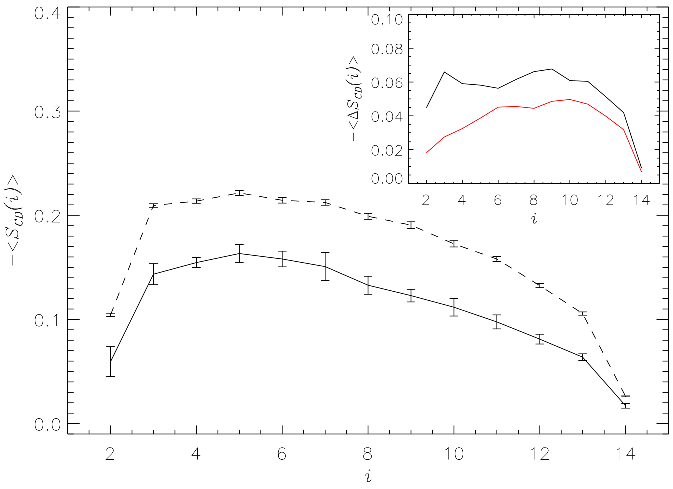

(b)

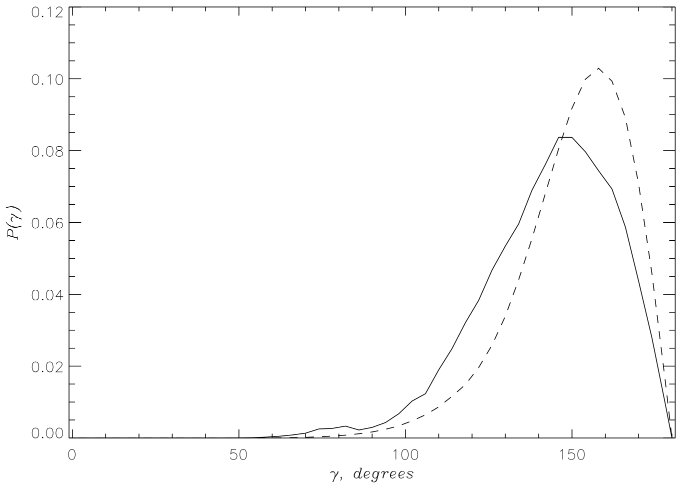

Fig. S5 (a) The lipid carbon-deuterium order parameter $-<S_{CD}(i)>$ computed for each carbon i in *sn-2* fatty acid tails. Sampling errors are shown by vertical bar lines. The inset shows the difference $-<\Delta S_{CD}(i)>$ between distant and

proximal -<$S_{CD}(i)$> values. Data for Aβ25-35 dimer and monomer are in black and red, respectively. (b) Probability distributions $P(\gamma)$ for lipid *sn-2* fatty acid tails to be tilted at an angle γ with respect to the bilayer normal. In both panels, solid and dashed lines refer to proximal and distant lipids.

Comparison of Aβ25-35 inserted dimers and monomers: Our simulations of Aβ25-35 dimers suggest that aggregation does not qualitatively change the secondary and tertiary structure of Aβ25-35 monomers. To provide a direct evidence, we plot in Fig. S6 the residue-specific distribution of helical structure in inserted Aβ25-35 **ID** dimers (Fig. 5) and inserted monomers **I** sampled in our previous REST simulations [2]. In both Aβ25-35 conformational ensembles the helical structure is localized in the C-terminus (<*H(R4)*>=0.60 in **ID** and 0.40 in **I**). Consistent with Fig. 2 this figure also indicates that aggregation stabilizes helical structure in Aβ25-35. In addition, Table S3 presents the list of stable intrapeptide contacts in these two Aβ25-35 species. It follows from the table that all five stable **ID** intrapeptide contacts are also present as stable interactions in **I**. Fig. S6 and Table S3 also include the data for the inserted monomers **IM** sampled in the dimer simulations (Fig. 5). As in the two ensembles discussed above **IM** features a stable helix in R4 (<*H(R4)*>=0.66) and four out of five stable **ID** contacts appear among stable intrapeptide **IM** interactions. It is also worth noting that **IM** and **I** ensembles share three most stable contacts in the same descending order of stability. Thus, Fig. S6 and Table S3 demonstrate that Aβ25-35 dimers utilize monomer-like peptide conformations, i.e., Aβ25-35 monomers inserted into the DMPC bilayer are aggregation-ready.

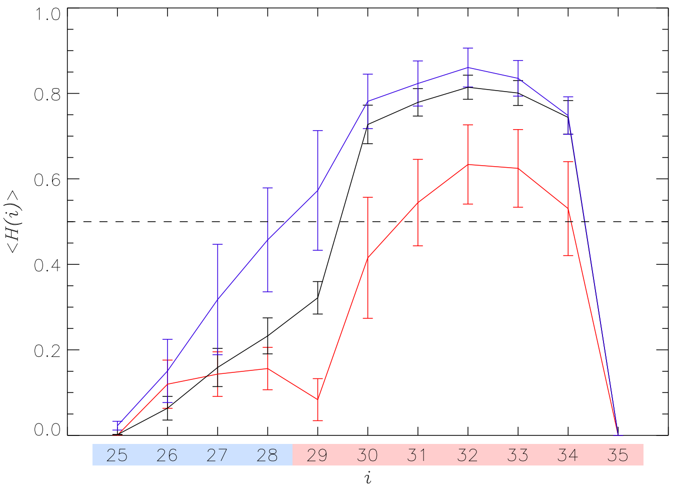

Figure S6 Helical propensities <*H(i)*> for amino acids *i* in Aβ25-35 dimers **ID** (in black), inserted monomers **I** from the previous study [2] (in red), and inserted monomers **IM** from the current study (in blue). Vertical bars show sampling errors. Regions R3-R4 are colored according to Fig. 1a.

Table S3 Stable[a] intrapeptide contacts in Aβ25-35 species.

**Aβ25-35 species**	**Rank**	***i***	***j***	**$<C(i,j)>$**
**ID** dimers	1	29	33	0.87
	2	29	32	0.87
	3	30	33	0.50
	4	32	35	0.41
	5	31	34	0.41
**I** monomers[b]	1	29	32	0.53
	2	29	33	0.52
	3	30	33	0.52
	4	31	34	0.46
	5	25	27	0.44
	6	32	35	0.41
**IM** monomers	1	29	32	0.85
	2	29	33	0.74
	3	30	33	0.57
	4	27	30	0.57
	5	31	34	0.52
	6	25	28	0.42
	7	26	28	0.41

[a]Contacts are stable if the probability of their occurrence $<C(i,j)>$>0.40.
[b]Aβ25-35 monomers inserted in the DMPC bilayer sampled in our previous REST simulations [2].

Effect of insertion depth on helical propensity: The helical propensity $<H(Z_m)>$ in Aβ25-35 peptide as a function of the position of its center of mass along the bilayer normal Z_m is presented in Fig. S7. It shows that in the peptides forming dimers $<H(Z_m)>$ steadily increases with the depth of their insertion in the bilayer. In contrast, the helical fraction remains largely unchanged, when Aβ25-35 monomer binds to the DMPC bilayer. The likely reason for the differing outcomes is an increase in the hydrophobic moment of **ID** dimer composed of two head-to-tail helices compared to a stand-alone monomer. Indeed, using the hydrophobic scale of Wimley et al [3], we found that the hydrophobic moments of **ID** and inserted monomer **IM** are 5.0 and 4.1 Å kcal/mol, respectively. Increase in the helix fraction with the insertion depth has also been observed by Garcia and coworkers for WALP-16 peptide [4].

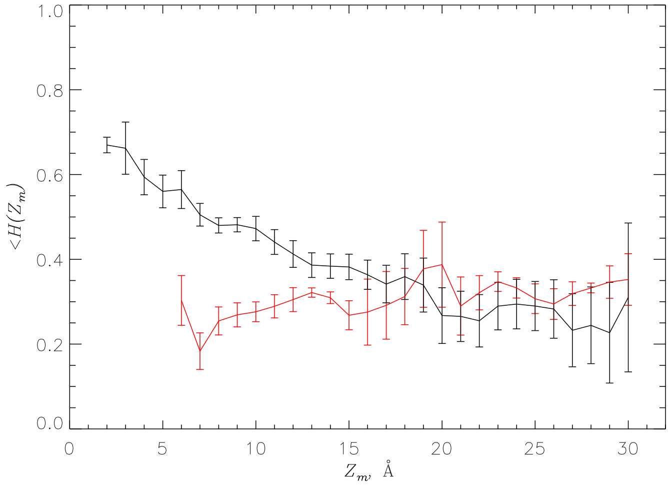

Figure S7 Helical propensities $<H(Z_m)>$ in Aβ25-35 dimers (in black) and monomers (in red, [2]) as a function of the position of peptide's center of mass along the bilayer normal Z_m. Vertical bars show sampling errors.

Possible impacts of bilayer composition and post-translational modifications on Aβ25-35 aggregation: It is important to discuss potential impact of bilayer composition on Aβ25-35 aggregation. The aggregation mechanism summarized in Figs. 5 and 6 is likely to remain valid as long as binding to the bilayer induces helical structure in the Aβ25-35 C-terminus. Although the precise details and the extent of this secondary structure transition depend on the bilayer composition, it was observed upon binding of Aβ peptides to diverse bilayers, including the zwitterionic (DMPC, current work and [2]), anionic (DMPS [5]), or cationic (DMPC + lipopeptides [6]) bilayers and the cholesterol-enriched DMPC bilayer [7]. Therefore, we hypothesize that the formation of **ID** dimer may be robust against changes in the bilayer composition. Interestingly, our preliminary data on Aβ25-35 monomer binding to the equimolar ternary bilayer composed of DMPC, PSM, and cholesterol support this conclusion by showing that binding to this bilayer promotes helical fraction approximately to the same extent as the pure DMPC bilayer [2]. It is important to note that the arguments presented above apply to Aβ25-35 dimers, whereas larger oligomers may respond differently to changes in bilayer composition.

It is interesting to discuss the question whether Aβ25-35 dimers promote permeation of Ca^{2+} ions. We have studied binding of Aβ10-40 monomers to the DMPC bilayer coincubated with 150 mM of Ca^{2+} ions [8]. That work showed that Ca^{2+} enhances binding affinity of anionic Aβ10-40 by disrupting the intrapeptide salt bridge and by making zwitterionic DMPC partially cationic due to strong coordination of Ca^{2+} with phosphate groups. In addition, anionic amino acids in Aβ10-40 attract Ca^{2+} ions bringing them with the peptide upon its shallow insertion into the bilayer. None of these factors are applicable to Aβ25-35, which is cationic and does not have intrapeptide salt-bridges. Therefore, it is conceivable that Ca^{2+} ions will decrease Aβ25-35 affinity to the bilayer consistent with the experimental findings of Tatulian and coworkers [9]. Hence, we tentatively conclude that Aβ25-35 dimer may not strongly affect Ca^{2+} permeation and larger aggregates embedded in the bilayer are needed to change this outcome. Importantly, the latter suggestion agrees well with the recent experiments showing that Ca^{2+} permeating pores are built of up to eight Aβ25-35 oligomers each involving from four to eight peptides [9] or formed by a single β-barrel oligomer composed of six Aβ25-35 peptides [10].

Finally, it is of interest to evaluate a possible effect of Ser26 phosphorylation on Aβ25-35. Previous studies have shown that this post-translational modification stabilizes Aβ1-40 oligomers and increases their cytotoxicity [11]. We are not aware of phosphorylation studies of Aβ25-35, but our current findings allow us to speculate on its plausible implications. Phosphorylation of Ser26 will introduce a negative charge in the peptide N-terminus. Since Ser26 is bound to the DMPC headgroups, we do not expect that phosphorylation will directly affect the location of this residue within the bilayer. Also, because Ser26 is not part of **ID** dimer aggregation interface, phosphorylation is unlikely to directly impact the **ID** species. However, phosphorylated Ser26 may form a salt-bridge with Lys28 as it was recently shown in replica exchange simulations of Aβ21-30 fragment [12]. If so, phosphorylation will indirectly compromise Lys28 binding to DMPC phosphate groups and, in turn, reduce Aβ25-35 propensity to insert into the bilayer thus shifting its conformational ensemble away from the inserted dimers. This hypothesis will be tested in our future simulations.

[1] Han, M. and Hansmann, U. H. E. (2011) Replica exchange molecular dynamics of the thermodynamics of fibril growth of Alzheimer's Aβ42 peptide. *J. Chem. Phys.* **135**, 065101.

[2] Smith, A. K. and Klimov, D. K. (2018) Binding of cytotoxic Aβ25-35 peptide to the DMPC lipid bilayer. *J. Chem. Inform. Model.* **58**, 1053–1065.

[3] Wimley, W. C., Creamer, T. P. and White, S. H. (1996) Solvation energies of amino acid side chains and backbone in a family of host-guest pentapeptides. Biochem. **35**, 5109–5124.
[4] Nymeyer, H., Woolf, T. B., and Garcia, A. E. (2005) Folding Is Not Required for Bilayer Insertion: Replica Exchange Simulations of an a-Helical Peptide with an Explicit Lipid Bilayer. Proteins: Structure, Function, and Bioinformatics **59**, 783–790.
[5] Lockhart, C. and Klimov, D. K. (2016) The Alzheimer's disease Aβ peptide binds to the anionic DMPS lipid bilayer. *Biochim. Biophys. Acta* **1858**, 1118–1128.
[6] Parikh, N. and Klimov, D. K. (2017) Inclusion of lipopeptides into the DMPC lipid bilayer prevents Abeta peptide insertion. *Phys. Chem. Chem. Phys.*, **19**, 10087-10098.
[7] Lockhart, C. and Klimov, D. K. (2017) Cholesterol changes the mechanism of Abeta peptide binding to the DMPC bilayer. *J. Chem. Inform. Model.*, **57**, 2554–2565.
[8] Lockhart, C. and Klimov, D.K. (2015) Calcium enhances binding of Abeta monomer to DMPC lipid bilayer. *Biophys. J.* **108**, 1807-1818.
[9] Kandel, N., Zheng, T., Huo, Q., and Tatulian, S. A. (2017) Membrane binding and pore formation by a cytotoxic fragment of amyloid β peptide. J. Phys. Chem. B **121**, 10293-10305.
[10] Kandel, N., Matos, J. O., and Tatulian, S. A. (2019) Structure of amyloid β25–35 in lipid environment and cholesterol dependent membrane pore formation. Sci. Reports **9**, 2689.
[11] Kumar, S., Wirths, O., Stüber, K, Wunderlich, P, Koch, P., Theil, S., Rezaei-Ghaleh, N., Zweckstetter5, M., Bayer, T. A., Brüstle, O., Thal, D. R., and Walter, J. (2016) Phosphorylation of the amyloid β-peptide at Ser26 stabilizes oligomeric assembly and increases neurotoxicity. Acta Neuropathol. **131**, 525–537.
[12] Rezaei-Ghaleh, N., Amininasab, M., Giller, K., Kumar, S., Stundl, A., Schneider, A., Becker, S., Walter, J., and Zweckstetter, M. (2014) Turn plasticity distinguishes different modes of amyloid-β aggregation. J. Amer. Chem. Soc. **136**, 4913−4919.
